# First Report of *Philometroides sanguineus* (Nematoda: Philometridae) in Farmed Goldfish (*Carassius auratus*) in Italy

**DOI:** 10.1111/jfd.14138

**Published:** 2025-04-27

**Authors:** Teresa Pirollo, Perla Tedesco, Marialetizia Fioravanti, Francesco Quaglio, Lorenzo Tarocchi, Enea Tentoni, Andrea Gustinelli

**Affiliations:** ^1^ Department of Veterinary Medical Sciences University of Bologna Bologna Italy; ^2^ Department of Comparative Biomedicine and Nutrition University of Padua Padova Italy; ^3^ APA‐CT Srl Forlì Italy

**Keywords:** biosecurity, *Carassius auratus*, goldfish, *Philometroides sanguineus*

## Abstract

The ornamental fish trade has facilitated the global dissemination of parasites, posing significant biosecurity risks. This study documents the first detection of the nematode *Philometroides sanguineus* in farmed goldfish ( *Carassius auratus* ) in Emilia‐Romagna, Italy. Specimens of gravid females, found in the caudal fins, and males, collected from the swim bladder, were identified through morphological, molecular and scanning electron microscope (SEM) analyses. This report underscores the risk of nonnative pathogen introduction via ornamental fish translocation, and the possibility of their spreading through the aquatic environment. In fact, the role of intermediate hosts, such as copepods, in parasite dissemination emphasises the need for stricter controls in systems reliant on surface water. Although *P. sanguineus* infection did not cause significant mortality in adult fish, its presence diminishes the ornamental value of infected specimens and raises concerns about the parasite's spread to native ecosystems. This case highlights the need to improve the biosecurity measures and comprehensive risk assessments to mitigate the introduction and propagation of alien parasites in aquaculture systems. Further research is necessary to evaluate the parasite's epidemiology, distribution and potential pathogenicity under different environmental and farming conditions.

## 1 Introduction

The ornamental fish culture and trade represent a sector of great economic interest on a global level, leading ornamental fish species to be one of the most translocated animals in the world (Evers et al. 2019; Ornamental Aquatic Trade Association Ltd. 2023).

In a scenario of growing demand for these fish and continuous drive for free trade, a frequent consequence of these movements is the accidental translocation of transmissible pathogens in geographical areas where they are not usually present, posing a substantial threat to the management of the consequences of their introduction and spread (Leprieur et al. 2009).

The accidental nature of pathogens’ release, specifically parasites, and the high likelihood of rapid spread through the translocation of fish or transport water make their dispersion and distribution challenging to regulate (Pegg et al. 2011).

Several examples of cointroduction of parasites in freshwater farmed fish in Europe are documented in the literature, each with varying degrees of impact on both the environment and the fish.

According to Esposito et al. (2023), the dissemination of a parasite can occur at three distinct spatial scales: (1) intercontinental scale, when parasites are translocated into Europe from extra‐European countries: for example, in Europe, the nonnative cestode *Schyzocotyle acheilognathi* spread through carp imported from China and far Eastern Russia to Western Russia and Eastern Europe, affecting farmed fish with high pathogenicity, especially in juveniles (Kuchta et al. 2018); Another notable example is the introduction of the parasite *Anguillicola crassus*, from infected Japanese eel imported from Taiwan into Germany during the 1980s (Kirk 2003); (2) intra‐European scale, involving the spread of parasites across different European regions, for example, the monogenean *Thaparocleidus vistulensis* associated with *Silurus glanis* (Galli et al. 2003; Paladini et al. 2008); and the cestode *Triaenophorus crassus* associated with *Esox lucius* (Gustinelli et al. 2006), both introduced from Central Europe to Italy; and (3) intracountry scale, when parasitised fish are moved from one body of water to another over a relatively short distance, as reported for example for the swim bladder nematode *Cystidicola farionis* (Gustinelli et al. 2008).

These examples of translocation emphasise the significance of studying the epidemiology of nonnative parasite species cointroduced with fish movements, whether via water or human activities, including commercial shipping, live animal trade or the deliberate release of unwanted pets into the wild as a ‘humane’ solution (Chan et al. 2019). The precautionary approach should be adopted to manage the risk posed to native fish populations by the unintentional introduction of alien pathogens, which are characterised not only by pronounced impacts, such as fish mortality, but also by sublethal and less evident effects. Furthermore, this approach should be expanded to include the control and protection of fish and aquatic environments that harbour nonnative pathogens, which, although less harmful, may still exert significant sublethal effects on fish populations (Pegg et al. 2011).

Goldfish, *Carassius auratus* Linnaeus, 1758 (Cypriniformes: Cyprinidae) is a freshwater cyprinid native to Eastern Asia and one of the most traded ornamental fish species worldwide, now farmed all over the world (Trujillo González et al. 2018). Due to its ability to spread spontaneously through hydrological networks after release of pet goldfish and/or unintentional introductions during stocking activities with juvenile common carp ( *Cyprinus carpio* ), this species has successfully established in lakes, rivers and reservoirs throughout Europe, North and South America, New Zealand and Australia (Carosi et al. 2019; Lorenzoni et al. 2010; Macchioni et al. 2015; Trujillo‐Gonzales et al. 2018). In Emilia Romagna region (Italy), there are several farms dedicated exclusively to ornamental fish, focusing particularly on varieties like Shubunkin, Canary (yellow), Sarasa (white and red) of the *C. auratus* species, as well as Koi carp (*C. rubrofuscus ‘koi’*), which hold a substantial economic value (Regazzi 2005).

Philometrids represent the most important group of dracunculoid nematodes infecting freshwater and marine teleosts worldwide and are mostly found in the body cavity and gonads of their hosts (Moravec and de Buron 2013). Certain species that infect freshwater fish, belonging to the genera *Philometra* and *Philometroides*, could be highly pathogenic to their hosts (Moravec and de Buron 2013). *Philometroides sanguineus* specifically infects *Carassius* spp. with pathogenic effects, particularly in juveniles of crucian carp ( *C. carassius* ), causing disease in both wild and cultured fish populations (Williams et al. 2012). In this regard, we aimed to draw attention to the first report in Italy of the presence of *P. sanguineus* (Nematoda, Philometridae) in goldfish ( *C. auratus* ) farmed in the Emilia‐Romagna region.

## 2 Materials and Methods

In March 2023, 10 adults of *C. auratus* (five alive and five dead) were sent to the Fish Pathology Unit (Department of Veterinary Medical Sciences—UNIBO) for parasitological analysis, after the field reports by fish health consultants indicating the presence of red worms in the tails of farmed goldfish. The fish, which were 3 years old, had been raised in ground tanks receiving surface water. According to the farmer's observations, less than 10% of the fish in the tank exhibited parasites on their tails. The live fish were sedated using MS‐222 (Tricaine Methanesulfonate or Ethyl 3‐aminobenzoate methanesulfonate), and the worms were extracted from the tail by applying gentle pressure. Dead fish were subjected to necropsy and to both external and internal parasitological examinations for the presence of any additional parasites.

The worms collected (five females, two males and five larvae L1) were preserved in 70% ethanol, clarified with Amman's lactophenol and subjected to morphological observation by light microscopy. Measurements were taken with the imaging software NIS‐Elements (Nikon, Campi Bisenzio [FI], Italy), and are given in micrometres, unless otherwise stated. The identification steps followed the key reported by Moravec (1994) and Moravec and de Buron (2013). Before clearing of two males and two females, a central piece of about 5 mm was cut for molecular analysis. Genomic DNA was extracted using PureLink Genomic DNA Kit (Life Technologies, Carlsbad, California) following the manufacturer's instructions, and the 18S rDNA amplified with the primers of Mariaux (1998) following the same protocol. The amplicons were purified by Nucleo‐Spin Gel and PCR Cleanup (Mackerey‐Nagel, Düren, Germany) and sequenced with an ABI 3730 DNA analyser (StarSEQ Mainz, Germany). The DNA trace files were assembled with ContigExpress (VectorNTI Advance 11 software, Invitrogen, Carlsbad, California) and the consensus sequences compared with previously published data by BLAST tools (https://blast.ncbi.nlm.nih.gov/Blast.cgi).

For SEM (scanning electron microscope) analysis, two formalin‐fixed females and one male were washed three times in phosphate buffer, postfixed in osmium tetroxide (1%), dehydrated in a graded ethanol series and dried in hexamethyldisilazane. Specimens were then mounted on aluminium stubs, sputter coated with gold–palladium and observed with a Phenom XL G2 Desktop SEM operating at 5 kV.

The caudal fin of two specimens infected by the gravid nematode female was dissected and preserved in 10% neutral buffered formalin for histopathological examination. After fixation, the tail was decalcified in 5% aqueous EDTA for 48 h and then processed for histology by staining 5‐μm‐thick sections with haematoxylin and eosin (H&E).

The sequences from this study have been deposited in GenBank under the accession numbers PV460257–58.

## 3 Results

**FIGURE 1:**
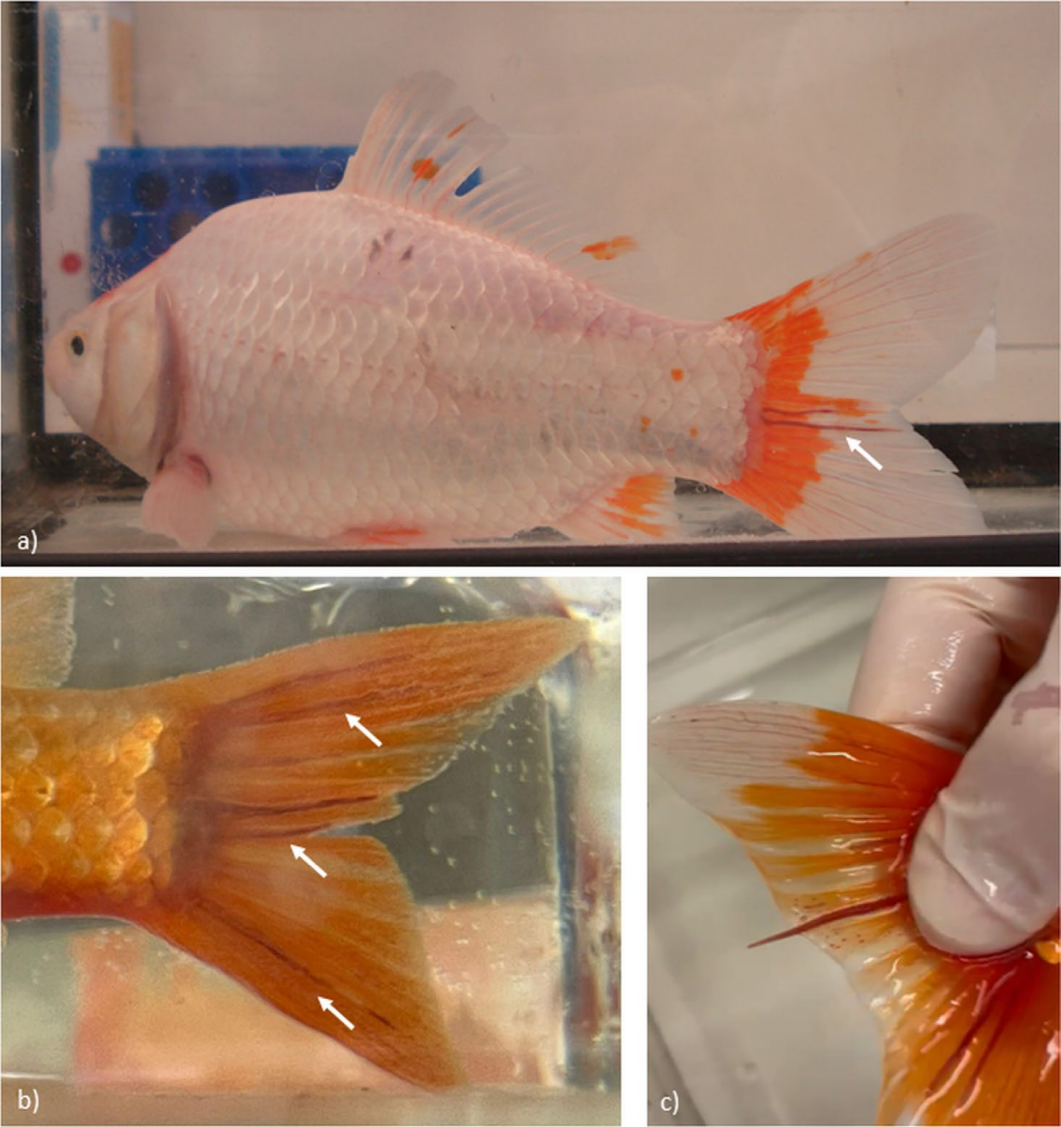
(a) Adult *Carassius auratus* infected with one female *P. sanguineus* , showing red‐coloured nematodes positioned in a U‐shape between caudal fin rays (arrow); (b) *Carassius auratus* tail infected with three gravid female *P. sanguineus* , showing nematodes within the same interray space (arrows); (c) detail of the tail of *Carassius auratus* at the moment of nematode extraction.

All fish showed the presence of one to three reddish round worms moving slowly between the bony rays of the caudal fin. Some of them were straight and others were arranged in ‘U‐shape’ with the ends directed towards the caudal margin of the fin (Figure 1). From the fish subjected to necropsy, two adult males were also found on the kidney and serosal surface of the swim bladder. The adults were thread‐like, 2–3 mm in length. A marked sexual dimorphism was observed, with large reddish gravid females located within the cutaneous tissue within the fin rays of the goldfish's tail (Figure 1).

The morphological features observed by light and scanning electron microscopy allowed us to identify all the nematodes as *Philometroides sanguineus*. Two specimens (one male and one female) were successfully amplified and sequenced. BLAST search gave 99.9% identity with *P. sanguineus* (DQ442676), confirming the morphological observations.

Below, the main morphometric characters of the isolated parasites are reported.

### 3.1 Gravid Female

**FIGURE 2:**
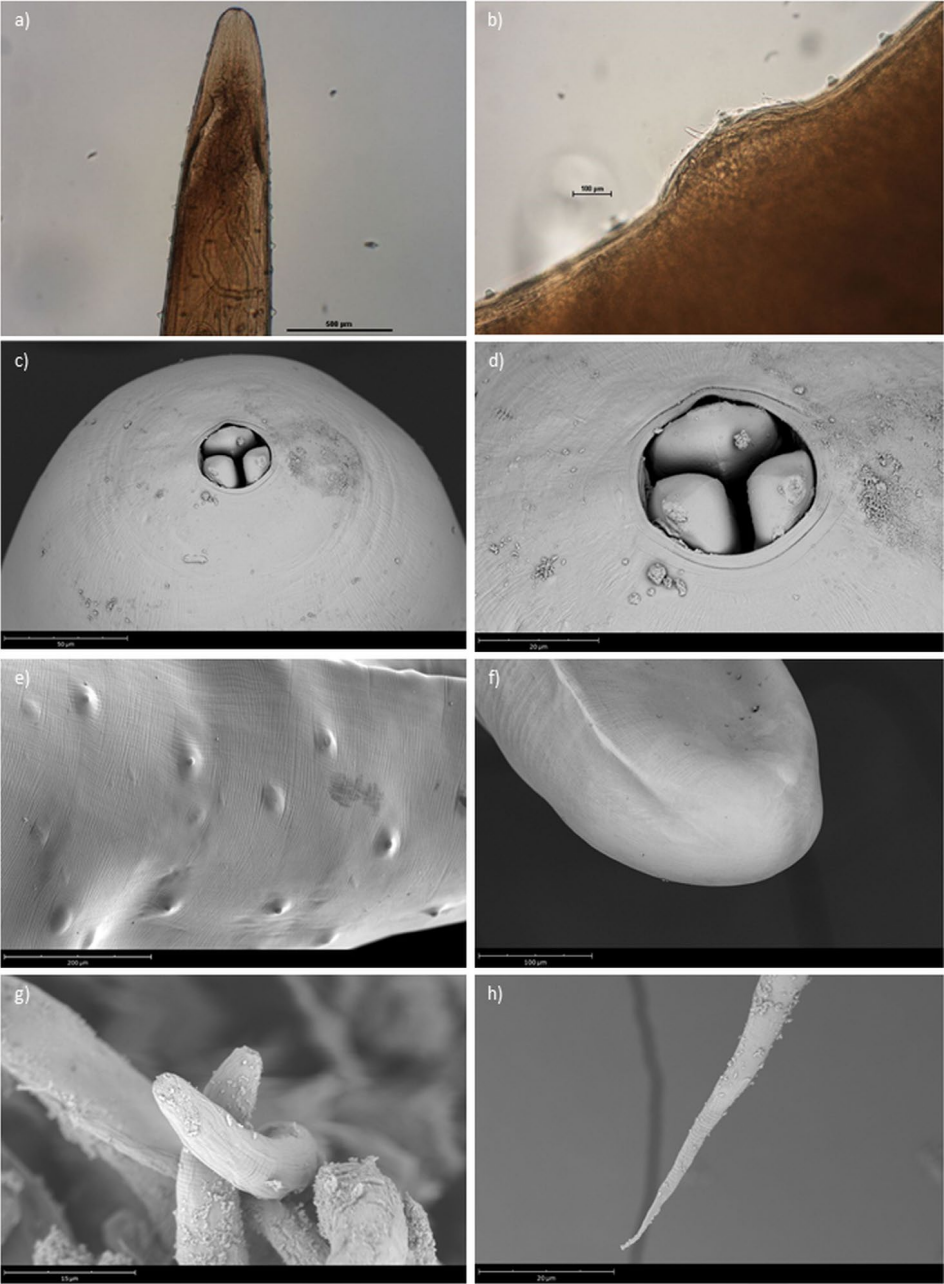
*Philometroides sanguineus* gravid female. (a) anterior end, lateral view (scale bar = 500 μm); (b) detail of vulva with protruding first stage larva (scale bar = 500 μm); (c) anterior end, subapical view (scale bar = 50 μm); (d) detail of oral aperture showing the anterior lobes of the three oesophageal sectors (scale bar = 20 μm); (e) detail of bosses over cuticle (scale bar = 200 μm); (f) posterior end (scale bar = 100 μm); (g) detail of first stage larva inside uterus (scale bar = 15 μm); (h) posterior end of first stage larva (scale bar = 20 μm).

Body reddish, elongate, cylindrical, narrowed and rounded at both ends (Figure 2a,f), 3.6–6 cm long, 0.9–1.1 mm wide; mouth opening circular, with visible anterior lobes of three oesophageal sectors (Figure 2b,c); cuticle smooth, with numerous bosses (Figure 2e); uterus filled with first stage larvae (Figure 2d,g,h); oesophagus narrow, muscular, swollen at anterior end, 3805.3 ± 621.7 (2986.3–4386.4) long, nerve ring encircling oesophagus, 298.5 ± 14.2 (282–314) from anterior end of body. Intestine brown to red, laterally displaced, narrowing towards posterior end. Anus, vagina and vulva atrophied. First stage larvae: body colourless, thin, 340.33 ± 10,8; (326.41–354.44) long; anterior end with minute dorsal tooth (Figure 2g); posterior end with elongated, sharply pointed tail (Figure 2h).

### 3.2 Adult Males

**FIGURE 3:**
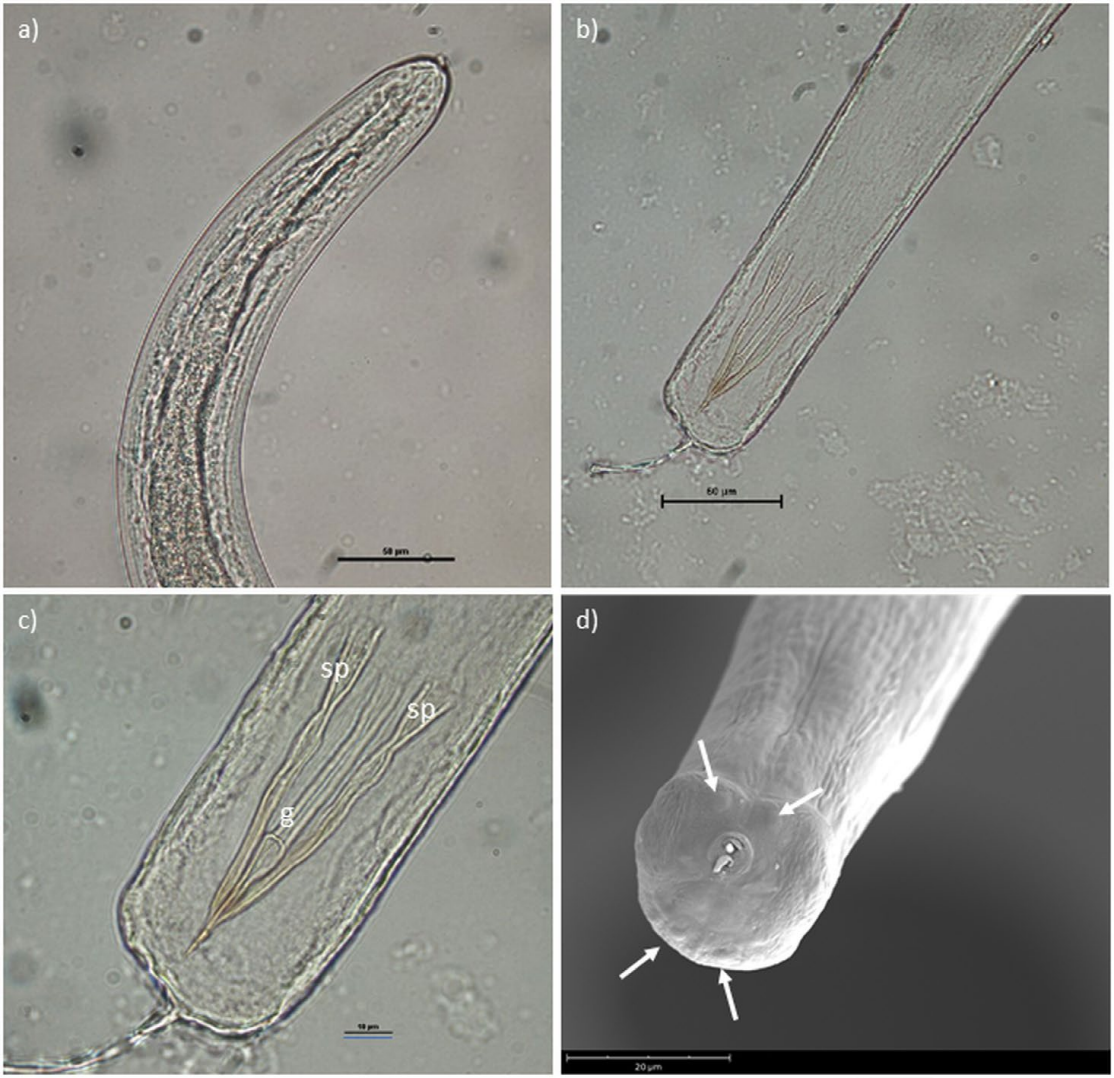
*Philometroides sanguineus* adult male. (a) anterior end, lateral view (scale bar = 50 μm); (b) posterior end, ventral view (scale bar = 60 μm); (c) detail of posterior end showing sclerotised spicules (sp) and gubernaculum (g) (scale bar = 10 μm); (d) detail of posterior end showing caudal papillae (arrows) (scale bar = 20 μm).

Body 2.95–3.15 mm long, 0.058–0.060 wide, with smooth cuticle; anterior end rounded (Figure 3a). Oesophagus cylindrical, slightly expanded at anterior end. Posterior end rounded, with two inconspicuous ventrolateral lobes and a larger dorsal lobe, two well sclerotised spicules, simple, smooth, subequal, with pointed tips, 0.069‐0.076 mm long, 0.050–0.053 mm wide (Figure 3b,d). Gubernaculum elongated, 0.069 mm long, with distal end bearing a small dorsal barb (Figure 3c).

**FIGURE 4:**
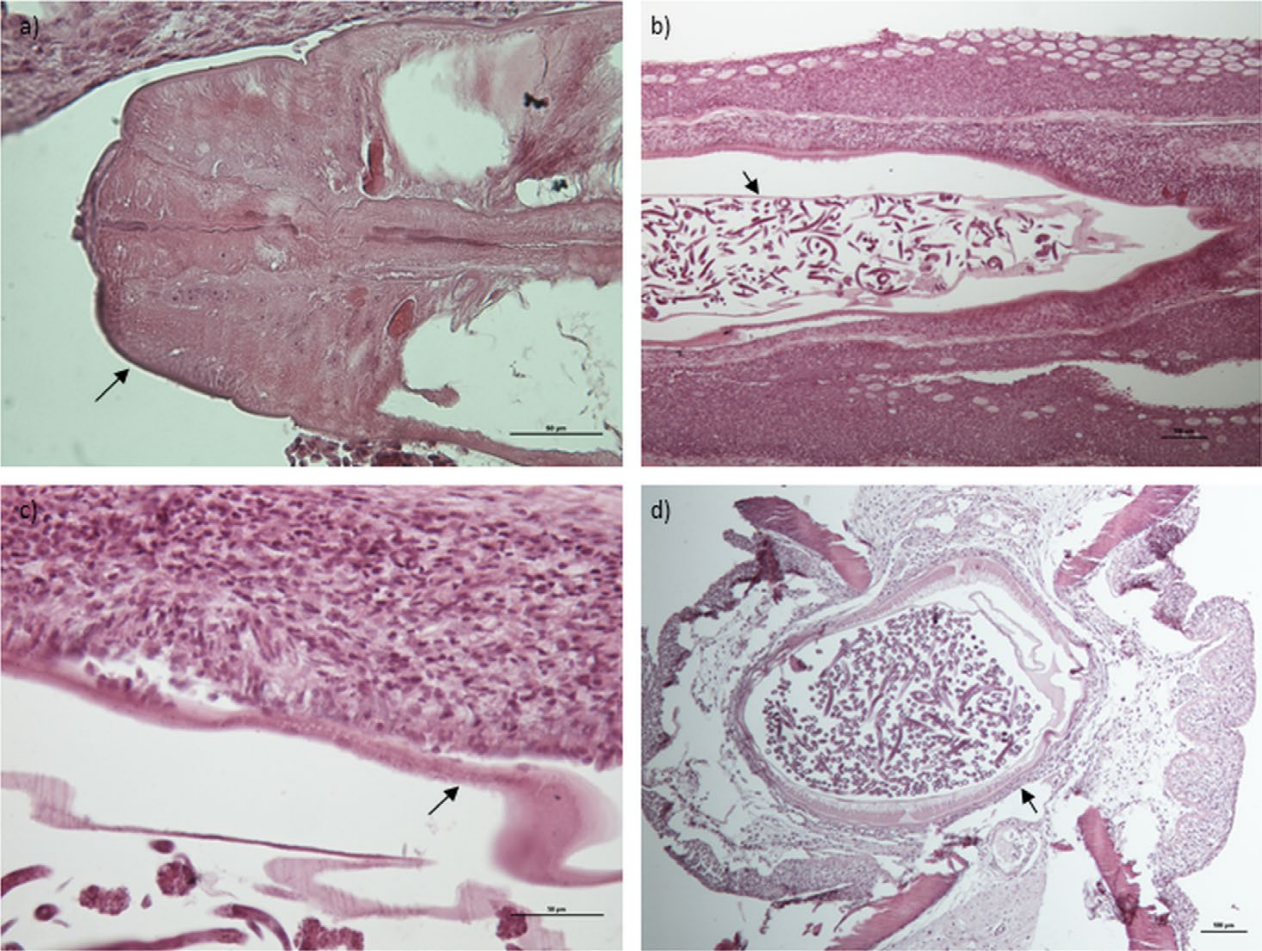
Histological sections of fin tail of goldfish infected by *P. sanguineus* (HE): (a) detail of the cephalic end of *P. sanguineus* (arrow) (scale bar = 50 μm); (b) longitudinal section of the nematode within the fin tail of goldfish surrounded by dermal mild inflammatory reaction; the parasite body shows inside the uterus (arrow) repleted by several larvae (scale bar = 100 μm); (c) detail at high magnification of the parasite cuticle (arrow) and external inflammatory reaction of the dermis with fibroblasts, lymphocytes and rare macrophages (scale bar = 50 μm); (d) transverse section of the goldfish fin tail, showing the parasite repleted by larvae (arrow) included between the adjacent lepidotrichia (scale bar = 100 μm).

At histology, the longitudinal sections of fin tail showed the presence of gravid females of nematodes referable to *P. sanguineus* between the adjacent fin rays (lepidotrichia) and within the dermal layer (Figure 4a,b). Inside the parasite body, a segment of uterus filled by larvae is evident (Figure 4b). The dermis around the parasites showed a focal mild inflammatory reaction consisting of lymphocytes, rare macrophages and neutrophils (Figure 4c). Specifically, in the outermost layer, fibroblasts were detected, while in the innermost layer, few lymphocytes and macrophages were evident. In the cross‐section, the interlepidotrichial connective tissue appeared deformed (stretched) by the body of the parasite (Figure 4d) with thickening of the epidermis between the two lepidotrichia. Unlike Williams et al. (2012), no eosinophilic granular cells were detected.

## 4 Discussion

Most species of *Philometroides* genus are found in Cypriniformes, inhabiting the freshwater systems of Eurasia (Stavrescu‐Bedivan and Vasile Scăeţeanu 2020). The first recorded case of *P. sanguineus* infection in Europe occurred in England in 1982, following a parasitological examination of a crucian carp from a farm pond in Essex (Pegg et al. 2011; Williams et al. 2012). Currently, this species is widespread across several European countries, including Sweden, Finland, Poland and other Eastern European states (Moravec 2023). Albeit spread in several European countries, to the best of our knowledge, no cases of *P. sanguineus* infection have been reported in Italy to date despite a consistent ornamental fish trade and the presence of numerous aquaculture facilities.

Even if this nematode is not considered a highly pathogenic parasite, some pathological changes induced by *P. sanguineus* have been linked to the seasonal migration of female worms within the host's tissue and their presence in the fins (Williams et al. 2012). Male and unfertilised female nematodes located in the swim bladder wall, as well as fertilised female dormant between migratory periods, cause only minor and localised changes (Moravec 1994). On the other hand, when large gravid females (up to 50 mm long) emerge to release larvae, a range of tissue responses (hyperplasia, acute inflammation, necrosis) and evident pathological changes (fin deformations, swelling of dorsal and caudal fin tissues, scale displacement) are triggered (Moravec and de Buron 2013).

Some previous studies have suggested that the severity of the damage is greatly influenced by the size and age of the host: fish less than 60 mm in length experience the most significant impact (Moravec 2023; Pegg et al. 2011; Williams et al. 2012).

In our study, adult goldfish harbouring the female showed no signs of debilitation, and the farmer did not report any losses of diseased fish, noticing the presence of the parasite only at the moment of selection and/or harvest. Moreover, histologically, the placement of the gravid female in the tail elicited only a relatively localised, mild inflammation response and compression of the vessel. However, it is important to note that the visible presence of the parasite in the fins diminishes the market and ornamental value of the fish.

In Italy, goldfish farming dates back to the last quarter of the 19th century, specifically in Emilia Romagna within the province of Bologna, where two companies started the production encouraged by the growing demand for this ornamental fish. According to a survey carried out in 2000, the Emilia Romagna region counts 24 farms exclusively dedicated to ornamental fish (Regazzi 2005). In the context of the current trade value of *C. auratus* in Italy, the risks posed by this parasite to the market of this ornamental fish may increase with its spread.

To understand the way of entry of the parasite into the Italian aquatic environments, it is important to possess a comprehensive knowledge of the parasite life cycle, the history of introductions and translocations of the fish host, the location of the farm and the source of water.

The life cycle of this parasite is seasonal, involving an intermediate host, typically ubiquitous free‐living copepods, becoming infected after ingesting free‐living first‐stage larvae (L1) released into the water by the gravid female. When these copepods, in which L1 reach the infective third stage (L3), are ingested by the fish definitive host, L3 will develop into adults. According to Moravec (2023), species of the superfamilies Cyclopoidea and Calanoidea are suitable intermediate hosts for the transmission of *P. sanguineus*, as demonstrated experimentally.

While male worms are consistently present in the body cavity of the fish host throughout the year (Williams et al. 2012), females migrate to the fins after fertilisation during autumn and winter, and by the end of May, the worm leaves the fins to release larvae into the waters through the process of ‘functional bursting’ (Pegg et al. 2011; Stavrescu‐Bedivan & Scăeţeanu Vasile 2020; Williams et al. 2012). Although assessing the route of an alien pathogen is challenging, knowledge on the life cycle of the parasite highlights potential pathways for its introduction into the farm environment.

In our case, two hypotheses may be considered: the introduction of infected fish from countries already affected by the parasite or the arrival of intermediate hosts/wild infected fish via the farm's water supply.

In the case under study, the farm follows a closed‐cycle system, which involves the selection of breeders and internal reproduction. As for the path of the incoming water to the fish farm, the goldfish farm is fed with surface water that could have been contaminated by upstream farms and sport fishing ponds, where crucian carps were present and frequently introduced from national and non‐Italian zones/countries. Consequently, the surface water may be the potential route for the introduction of this alien parasite or of copepods infected by *P. sanguineus* L3.

In general, even though species introduced for aquaculture/sale purposes should be maintained under conditions preventing their escape, parasites could still be disseminated via water currents, potentially exposing endemic species in the surrounding area (e.g., to a downstream farm) (Peeler et al. 2011).

As observed in the case of *Anguillicola crassus*, a nematode originating from Asia and now widespread across Europe, including Italy, the role of intermediate host has been important in its dissemination, similar to *P. sanguineus* . This nematode uses ubiquitous copepods as intermediate hosts, which facilitates its spread through various water systems, impacting both farmed and wild European eels (Giari et al. 2021). Although direct cycle parasites are favoured in the possibility of establishing themselves and becoming invasive, these cases exemplify how intermediate hosts, when ubiquitous in the aquatic environment, can significantly contribute to the spread of parasites with complex biological life cycles (Lymbery et al. 2014).

Given that *P. sanguineus* also utilises several species of copepods in its life cycle and considering the ubiquity of the copepod species involved in the cycle, it is plausible to suspect their role in the introduction and spread of the parasite. In this context, it would be critical to include this risk factor in a practical and effective risk management protocol to assess the pathways of entry and the potential dangers posed by nonnative pathogens to aquatic animals.

In literature, different examples can be found at different geographical levels. For instance, at a global level, the Aquatic Animal Health Code (WOAH 2023) includes a chapter dedicated to import risk analysis and sections addressing trade measures, importation/exportation procedures and health certification. Nevertheless, the primary focus remains on notifiable diseases listed in the Aquatic Code and mostly related to viral aetiology (among the parasitic diseases, only Gyrodactylosis by *Gyrodactylus salaris* is still listed).

At the European level, the EU Regulation 2016/429 (also known as Animal Health Law) contains sections related to disease prevention and preparation for potential outbreaks, detailing biosecurity measures, such as the use of diagnostic tools, vaccination and medical treatments. However, similar to the global framework, this regulation predominantly addresses viral notifiable diseases, overlooking the potential issues related to the spread of parasites that may not cause sudden and/or abnormal mortalities but could have a long‐term impact on fish health and biodiversity.

One interesting model for risk management of nonnative pathogens in aquatic environments is the European Non‐native Species in Aquaculture Risk Analysis Scheme (ENSARS), developed to comply with Regulation (EC) No. 708/2007 concerning the use of alien and locally absent species in aquaculture. ENSARS is designed to identify and evaluate potential risks associated with nonnative species in aquaculture, including parasites and the subsequent ecological and socio‐economic impacts (Copp et al. 2016).

Furthermore, ENSARS emphasises the importance of ongoing risk assessment and communication with stakeholders, including farmers, to effectively mitigate risks.

By adopting such a proactive and structured risk management scheme, countries can better safeguard their aquatic ecosystems from the potential threats posed by nonnative pathogens, ensuring sustainable aquaculture practices and protecting biodiversity (Copp et al. 2016).

This work represents the first report of *P. sanguineus* in farmed goldfish from Italy. Based on gross and histopathological observations, the infection appears to have no severe pathological implications for the fish. Nevertheless, future work should focus on its epidemiology and current distribution within the Italian territory, introduction pathways and risks related to its spread. Additionally, the pathogenic role in smaller fish and under different farming conditions needs further study. It is well known that parasite invasions can be unpredictable and often lack sufficient information for a reliable assessment or modelling of disease risk (Williams et al. 2013). However, this paper highlights the necessity to implement risk assessment protocols against the introduction of alien parasitic species.

## Author Contributions

**Teresa Pirollo:** conceptualization, investigation, writing – original draft, methodology, writing – review and editing, data curation, validation. **Perla Tedesco:** conceptualization, investigation, writing – original draft, writing – review and editing, methodology, validation, data curation. **Marialetizia Fioravanti:** conceptualization, investigation, writing – original draft, project administration, supervision, data curation, writing – review and editing, methodology, validation, visualization. **Francesco Quaglio:** conceptualization, investigation, writing – review and editing, methodology, data curation. **Lorenzo Tarocchi:** methodology, writing – review and editing, formal analysis. **Enea Tentoni:** methodology, writing – review and editing, formal analysis. **Andrea Gustinelli:** conceptualization, investigation, writing – original draft, writing – review and editing, data curation, supervision, project administration, methodology, validation.

## Conflicts of Interest

The authors declare no conflicts of interest.

## Data Availability

The data that support the findings of this study are available from the corresponding author upon reasonable request.
